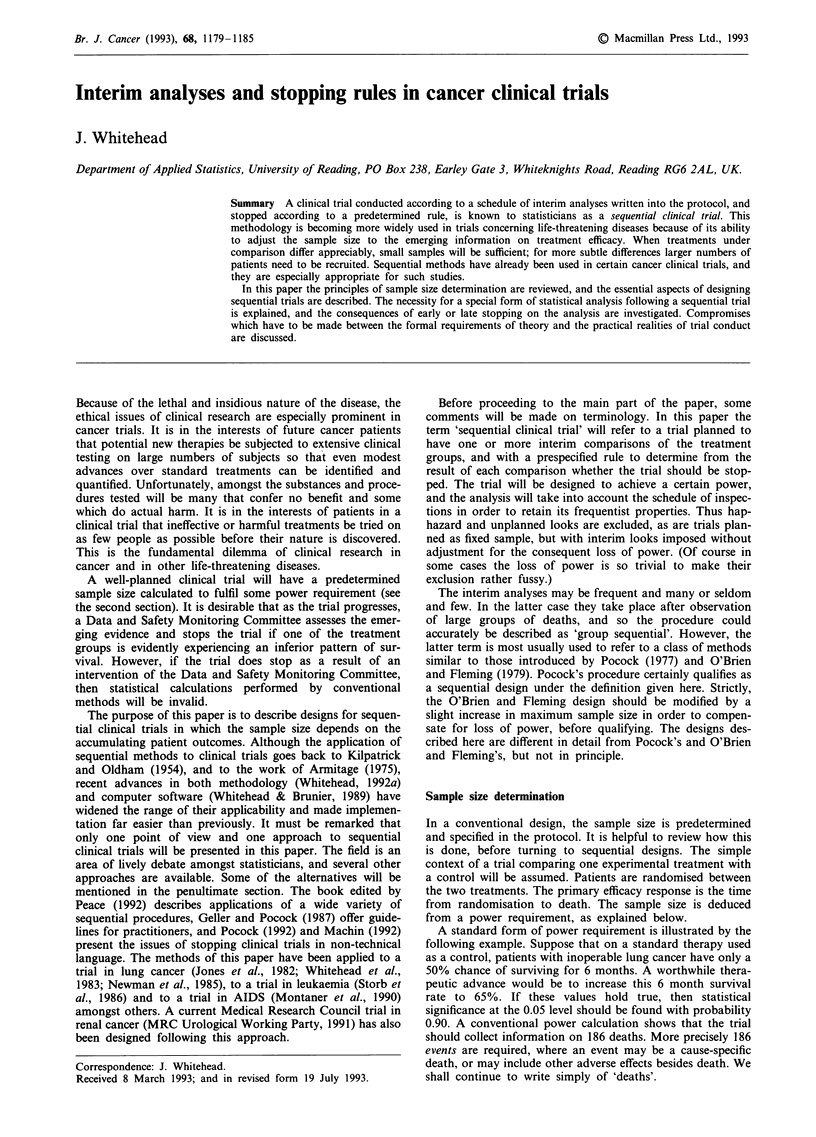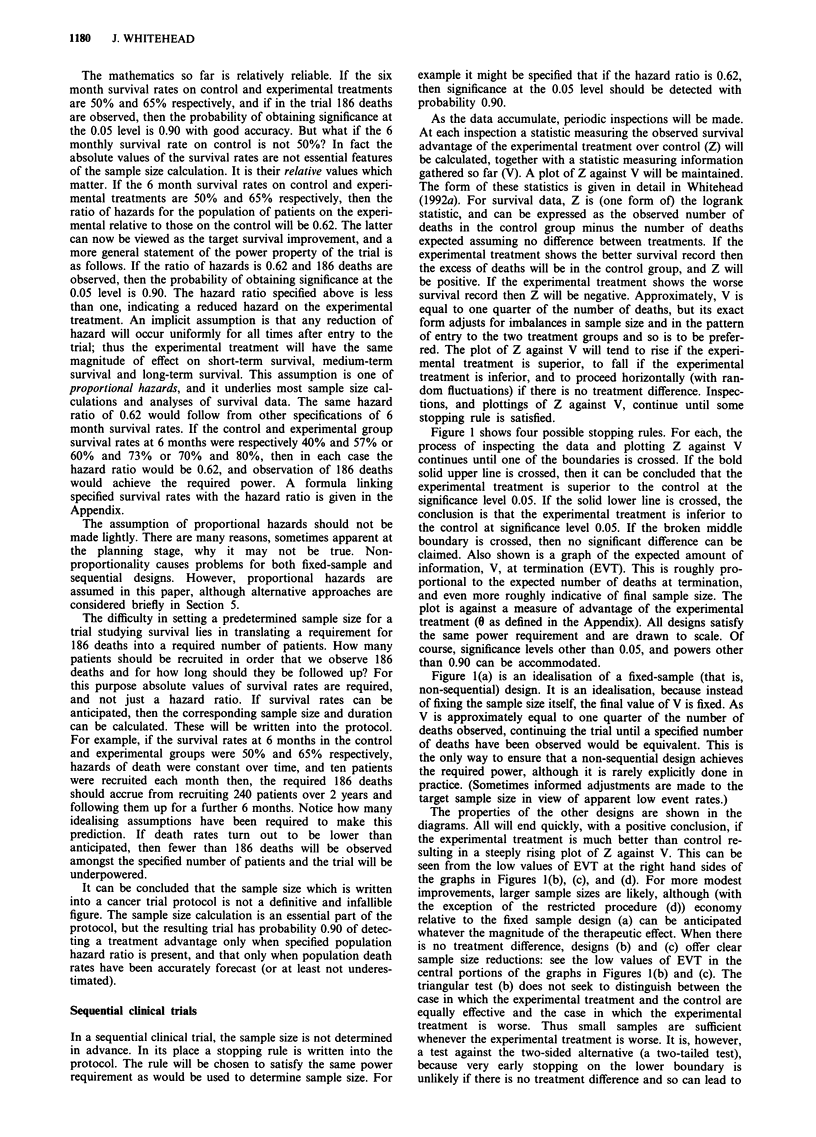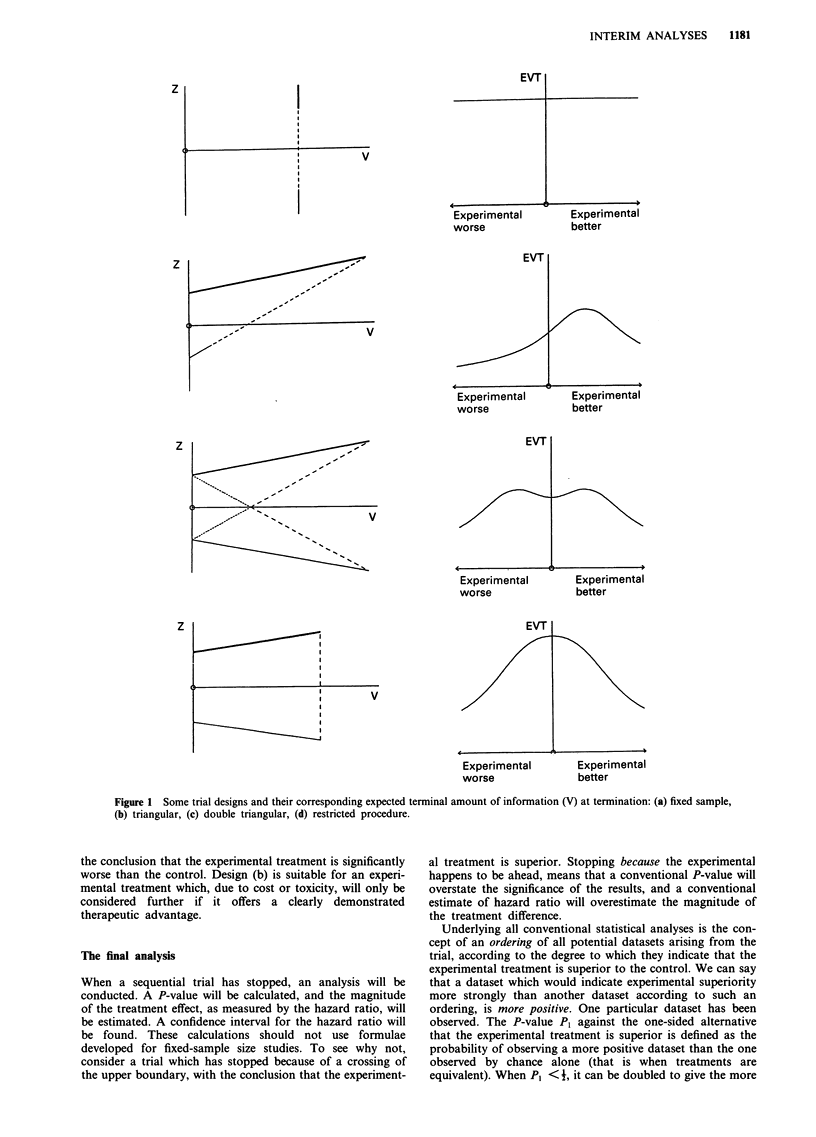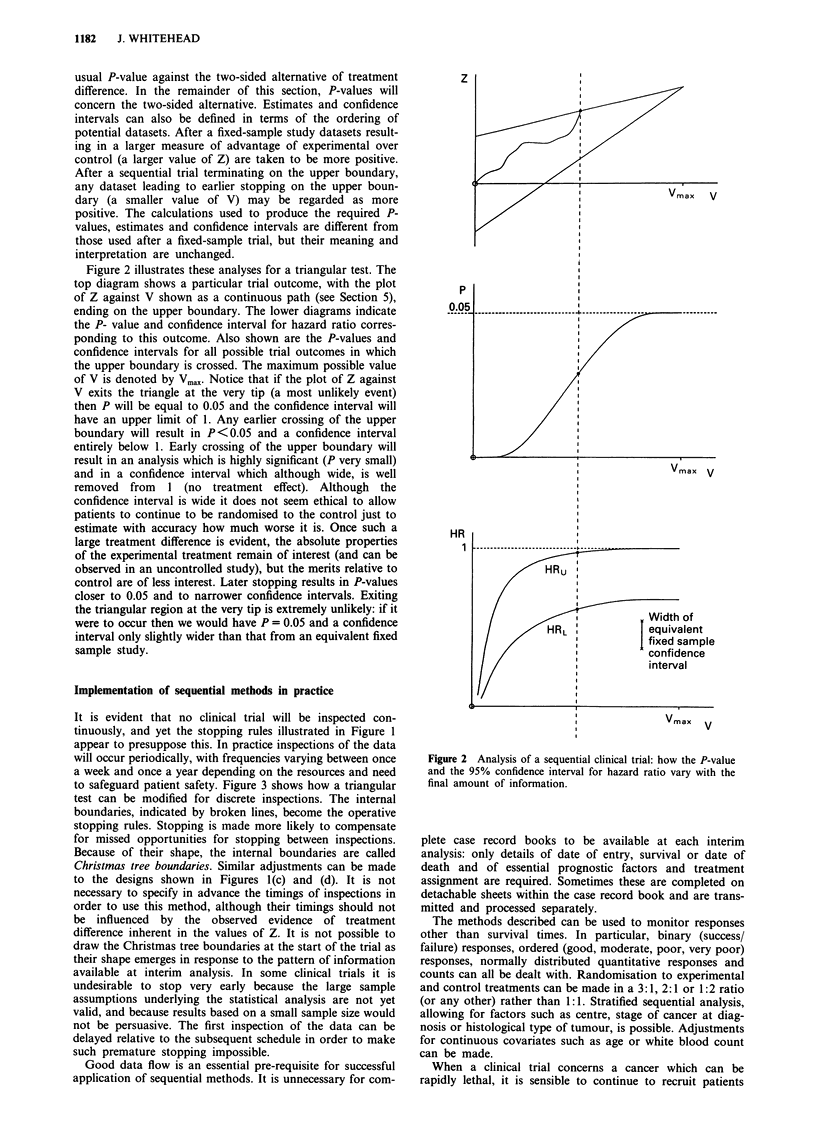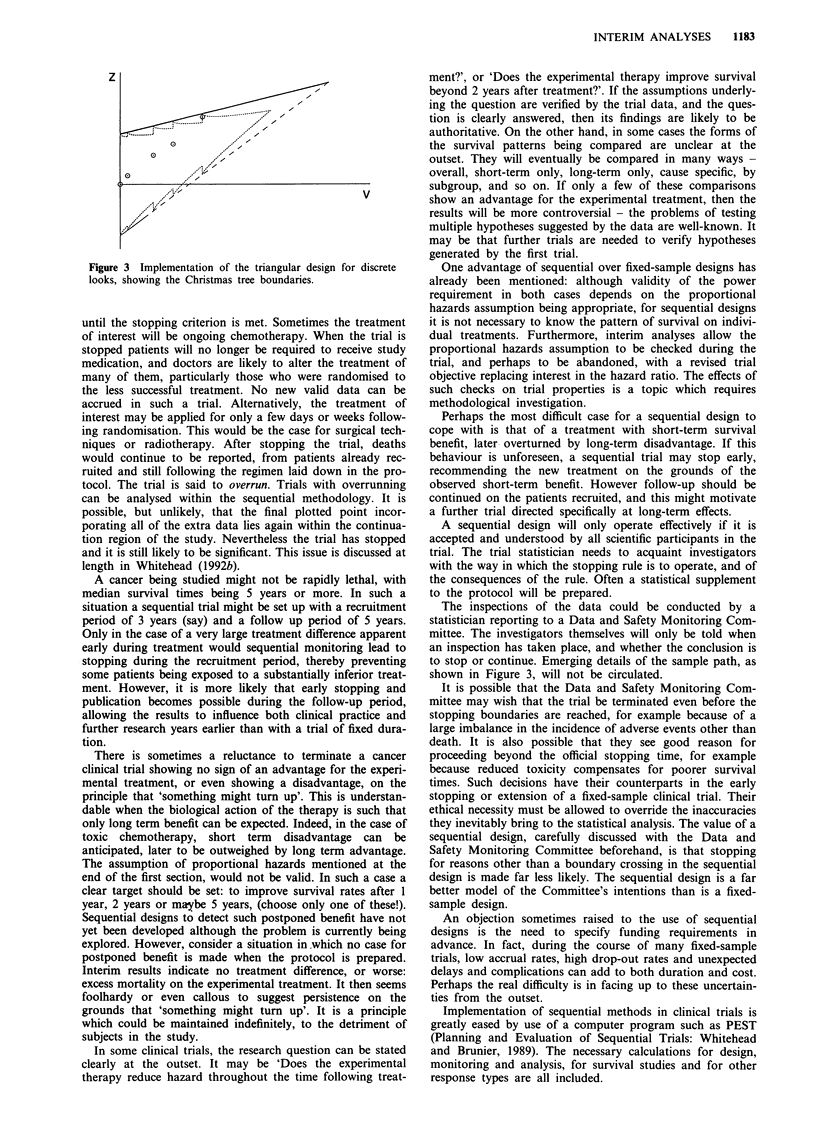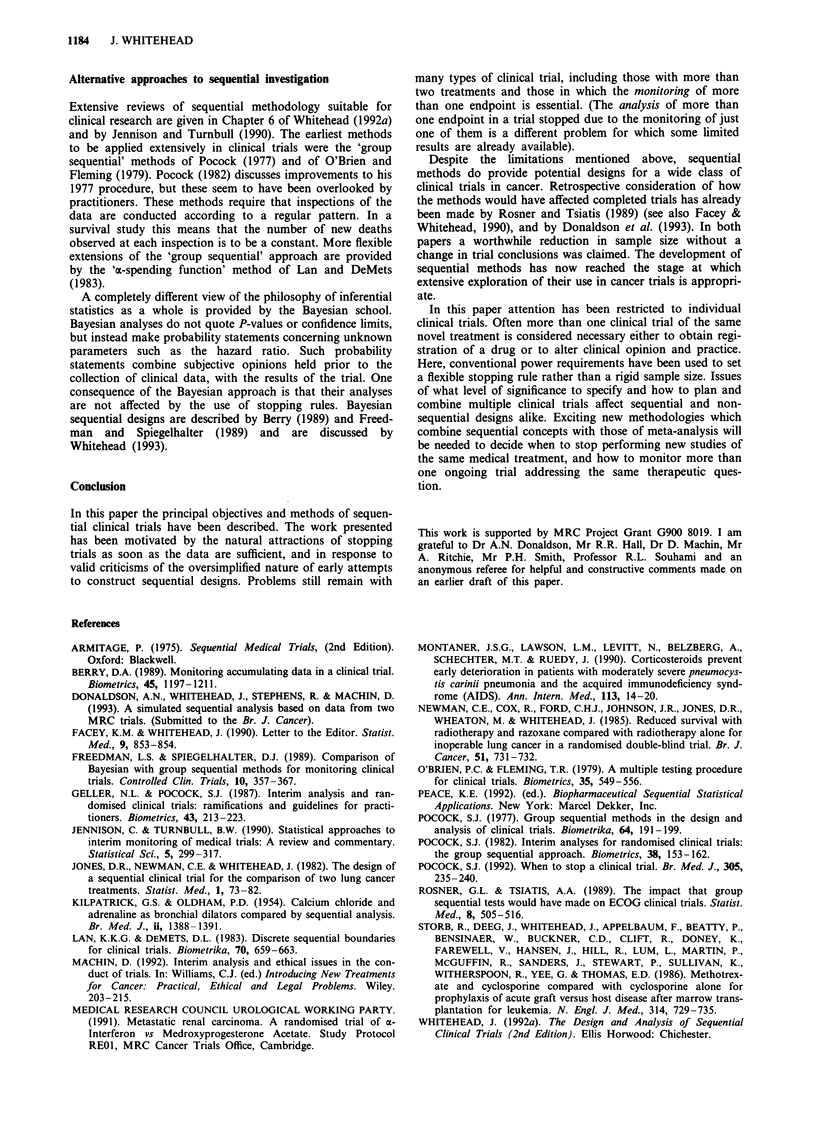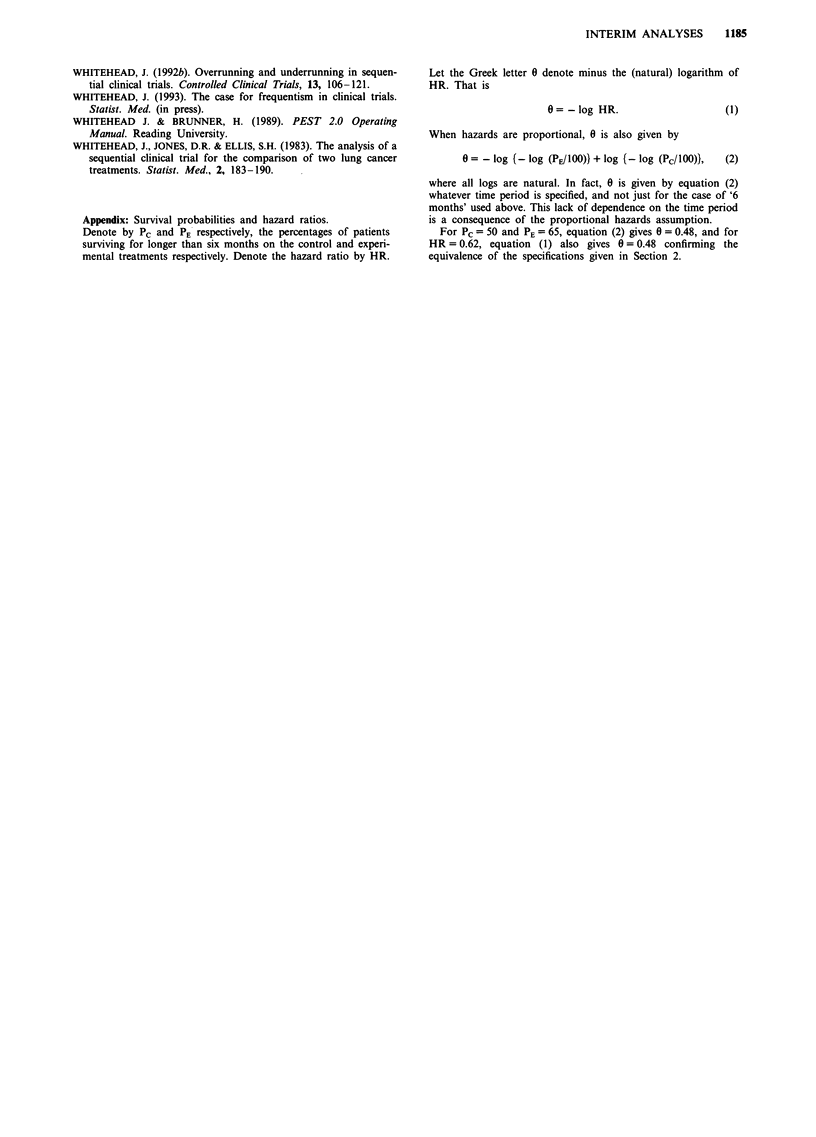# Interim analyses and stopping rules in cancer clinical trials.

**DOI:** 10.1038/bjc.1993.500

**Published:** 1993-12

**Authors:** J. Whitehead

**Affiliations:** Department of Applied Statistics, University of Reading, UK.

## Abstract

A clinical trial conducted according to a schedule of interim analyses written into the protocol, and stopped according to a predetermined rule, is known to statisticians as a sequential clinical trial. This methodology is becoming more widely used in trials concerning life-threatening diseases because of its ability to adjust the sample size to the emerging information on treatment efficacy. When treatments under comparison differ appreciably, small samples will be sufficient; for more subtle differences larger numbers of patients need to be recruited. Sequential methods have already been used in certain cancer clinical trials, and they are especially appropriate for such studies. In this paper the principles of sample size determination are reviewed, and the essential aspects of designing sequential trials are described. The necessity for a special form of statistical analysis following a sequential trial is explained, and the consequences of early or late stopping on the analysis are investigated. Compromises which have to be made between the formal requirements of theory and the practical realities of trial conduct are discussed.


Br. J. Cancer (1993), 68, 1179-1185 © Macmillan Press Ltd., 1993

### Interim analyses and stopping rules in cancer clinical trials

J. Whitehead

*Department of Applied Statistics, University of Reading, PO Box 238, Earley Gate 3, Whiteknights Road, Reading RG6 2AL, UK.*

**Summary** A clinical trial conducted according to a schedule of interim analyses written into the protocol, and stopped according to a predetermined rule, is known to statisticians as a *sequential clinical trial.* This methodology is becoming more widely used in trials concerning life-threatening diseases because of its ability to adjust the sample size to the emerging information on treatment efficacy. When treatments under comparison differ appreciably, small samples will be sufficient; for more subtle differences larger numbers of patients need to be recruited. Sequential methods have already been used in certain cancer clinical trials, and they are especially appropriate for such studies.

In this paper the principles of sample size determination are reviewed, and the essential aspects of designing sequential trials are described. The necessity for a special form of statistical analysis following a sequential trial is explained, and the consequences of early or late stopping on the analysis are investigated. Compromises which have to be made between the formal requirements of theory and the practical realities of trial conduct are discussed.

Because of the lethal and insidious nature of the disease, the ethical issues of clinical research are especially prominent in cancer trials. It is in the interests of future cancer patients that potential new therapies be subjected to extensive clinical testing on large numbers of subjects so that even modest advances over standard treatments can be identified and quantified. Unfortunately, amongst the substances and procedures tested will be many that confer no benefit and some which do actual harm. It is in the interests of patients in a clinical trial that ineffective or harmful treatments be tried on as few people as possible before their nature is discovered. This is the fundamental dilemma of clinical research in cancer and in other life-threatening diseases.

A well-planned clinical trial will have a predetermined sample size calculated to fulfil some power requirement (see the second section). It is desirable that as the trial progresses, a Data and Safety Monitoring Committee assesses the emerging evidence and stops the trial if one of the treatment groups is evidently experiencing an inferior pattern of survival. However, if the trial does stop as a result of an intervention of the Data and Safety Monitoring Committee, then statistical calculations performed by conventional methods will be invalid.

The purpose of this paper is to describe designs for sequential clinical trials in which the sample size depends on the accumulating patient outcomes. Although the application of sequential methods to clinical trials goes back to Kilpatrick and Oldham (1954), and to the work of Armitage (1975), recent advances in both methodology (Whitehead, 1992*a*) and computer software (Whitehead & Brunier, 1989) have widened the range of their applicability and made implementation far easier than previously. It must be remarked that only one point of view and one approach to sequential clinical trials will be presented in this paper. The field is an area of lively debate amongst statisticians, and several other approaches are available. Some of the alternatives will be mentioned in the penultimate section. The book edited by Peace (1992) describes applications of a wide variety of sequential procedures, Geller and Pocock (1987) offer guidelines for practitioners, and Pocock (1992) and Machin (1992) present the issues of stopping clinical trials in non-technical language. The methods of this paper have been applied to a trial in lung cancer (Jones *et al.*, 1982; Whitehead *et al.*, 1983; Newman *et al.*, 1985), to a trial in leukaemia (Storb *et al.*, 1986) and to a trial in AIDS (Montaner *et al.*, 1990) amongst others. A current Medical Research Council trial in renal cancer (MRC Urological Working Party, 1991) has also been designed following this approach.

Correspondence: J. Whitehead.
Received 8 March 1993; and in revised form 19 July 1993.

Before proceeding to the main part of the paper, some comments will be made on terminology. In this paper the term 'sequential clinical trial' will refer to a trial planned to have one or more interim comparisons of the treatment groups, and with a prespecified rule to determine from the result of each comparison whether the trial should be stopped. The trial will be designed to achieve a certain power, and the analysis will take into account the schedule of inspections in order to retain its frequentist properties. Thus haphazard and unplanned looks are excluded, as are trials planned as fixed sample, but with interim looks imposed without adjustment for the consequent loss of power. (Of course in some cases the loss of power is so trivial to make their exclusion rather fussy.)

The interim analyses may be frequent and many or seldom and few. In the latter case they take place after observation of large groups of deaths, and so the procedure could accurately be described as 'group sequential'. However, the latter term is most usually used to refer to a class of methods similar to those introduced by Pocock (1977) and O'Brien and Fleming (1979). Pocock's procedure certainly qualifies as a sequential design under the definition given here. Strictly, the O'Brien and Fleming design should be modified by a slight increase in maximum sample size in order to compensate for loss of power, before qualifying. The designs described here are different in detail from Pocock's and O'Brien and Fleming's, but not in principle.

### Sample size determination

In a conventional design, the sample size is predetermined and specified in the protocol. It is helpful to review how this is done, before turning to sequential designs. The simple context of a trial comparing one experimental treatment with a control will be assumed. Patients are randomised between the two treatments. The primary efficacy response is the time from randomisation to death. The sample size is deduced from a power requirement, as explained below.

A standard form of power requirement is illustrated by the following example. Suppose that on a standard therapy used as a control, patients with inoperable lung cancer have only a 50% chance of surviving for 6 months. A worthwhile therapeutic advance would be to increase this 6 month survival rate to 65%. If these values hold true, then statistical significance at the 0.05 level should be found with probability 0.90. A conventional power calculation shows that the trial should collect information on 186 deaths. More precisely 186 *events* are required, where an event may be a cause-specific death, or may include other adverse effects besides death. We shall continue to write simply of 'deaths'.

The mathematics so far is relatively reliable. If the six month survival rates on control and experimental treatments are 50% and 65% respectively, and if in the trial 186 deaths are observed, then the probability of obtaining significance at the 0.05 level is 0.90 with good accuracy. But what if the 6 monthly survival rate on control is not 50%? In fact the absolute values of the survival rates are not essential features of the sample size calculation. It is their *relative* values which matter. If the 6 month survival rates on control and experimental treatments are 50% and 65% respectively, then the ratio of hazards for the population of patients on the experimental relative to those on the control will be 0.62. The latter can now be viewed as the target survival improvement, and a more general statement of the power property of the trial is as follows. If the ratio of hazards is 0.62 and 186 deaths are observed, then the probability of obtaining significance at the 0.05 level is 0.90. The hazard ratio specified above is less than one, indicating a reduced hazard on the experimental treatment. An implicit assumption is that any reduction of hazard will occur uniformly for all times after entry to the trial; thus the experimental treatment will have the same magnitude of effect on short-term survival, medium-term survival and long-term survival. This assumption is one of *proportional hazards*, and it underlies most sample size calculations and analyses of survival data. The same hazard ratio of 0.62 would follow from other specifications of 6 month survival rates. If the control and experimental group survival rates at 6 months were respectively 40% and 57% or 60% and 73% or 70% and 80%, then in each case the hazard ratio would be 0.62, and observation of 186 deaths would achieve the required power. A formula linking specified survival rates with the hazard ratio is given in the Appendix.

The assumption of proportional hazards should not be made lightly. There are many reasons, sometimes apparent at the planning stage, why it may not be true. Non-proportionality causes problems for both fixed-sample and sequential designs. However, proportional hazards are assumed in this paper, although alternative approaches are considered briefly in Section 5.

The difficulty in setting a predetermined sample size for a trial studying survival lies in translating a requirement for 186 deaths into a required number of patients. How many patients should be recruited in order that we observe 186 deaths and for how long should they be followed up? For this purpose absolute values of survival rates are required, and not just a hazard ratio. If survival rates can be anticipated, then the corresponding sample size and duration can be calculated. These will be written into the protocol. For example, if the survival rates at 6 months in the control and experimental groups were 50% and 65% respectively, hazards of death were constant over time, and ten patients were recruited each month then, the required 186 deaths should accrue from recruiting 240 patients over 2 years and following them up for a further 6 months. Notice how many idealising assumptions have been required to make this prediction. If death rates turn out to be lower than anticipated, then fewer than 186 deaths will be observed amongst the specified number of patients and the trial will be underpowered.

It can be concluded that the sample size which is written into a cancer trial protocol is not a definitive and infallible figure. The sample size calculation is an essential part of the protocol, but the resulting trial has probability 0.90 of detecting a treatment advantage only when specified population hazard ratio is present, and that only when population death rates have been accurately forecast (or at least not underestimated).

### Sequential clinical trials

In a sequential clinical trial, the sample size is not determined in advance. In its place a stopping rule is written into the protocol. The rule will be chosen to satisfy the same power requirement as would be used to determine sample size. For example it might be specified that if the hazard ratio is 0.62, then significance at the 0.05 level should be detected with probability 0.90.

As the data accumulate, periodic inspections will be made. At each inspection a statistic measuring the observed survival advantage of the experimental treatment over control (Z) will be calculated, together with a statistic measuring information gathered so far (V). A plot of Z against V will be maintained. The form of these statistics is given in detail in Whitehead (1992*a*). For survival data, Z is (one form of) the logrank statistic, and can be expressed as the observed number of deaths in the control group minus the number of deaths expected assuming no difference between treatments. If the experimental treatment shows the better survival record then the excess of deaths will be in the control group, and Z will be positive. If the experimental treatment shows the worse survival record then Z will be negative. Approximately, V is equal to one quarter of the number of deaths, but its exact form adjusts for imbalances in sample size and in the pattern of entry to the two treatment groups and so is to be preferred. The plot of Z against V will tend to rise if the experimental treatment is superior, to fall if the experimental treatment is inferior, and to proceed horizontally (with random fluctuations) if there is no treatment difference. Inspections, and plottings of Z against V, continue until some stopping rule is satisfied.

Figure 1 shows four possible stopping rules. For each, the process of inspecting the data and plotting Z against V continues until one of the boundaries is crossed. If the bold solid upper line is crossed, then it can be concluded that the experimental treatment is superior to the control at the significance level 0.05. If the solid lower line is crossed, the conclusion is that the experimental treatment is inferior to the control at significance level 0.05. If the broken middle boundary is crossed, then no significant difference can be claimed. Also shown is a graph of the expected amount of information, V, at termination (EVT). This is roughly proportional to the expected number of deaths at termination, and even more roughly indicative of final sample size. The plot is against a measure of advantage of the experimental treatment ($\theta$ as defined in the Appendix). All designs satisfy the same power requirement and are drawn to scale. Of course, significance levels other than 0.05, and powers other than 0.90 can be accommodated.

Figure 1(a) is an idealisation of a fixed-sample (that is, non-sequential) design. It is an idealisation, because instead of fixing the sample size itself, the final value of V is fixed. As V is approximately equal to one quarter of the number of deaths observed, continuing the trial until a specified number of deaths have been observed would be equivalent. This is the only way to ensure that a non-sequential design achieves the required power, although it is rarely explicitly done in practice. (Sometimes informed adjustments are made to the target sample size in view of apparent low event rates.)

The properties of the other designs are shown in the diagrams. All will end quickly, with a positive conclusion, if the experimental treatment is much better than control resulting in a steeply rising plot of Z against V. This can be seen from the low values of EVT at the right hand sides of the graphs in Figures 1(b), (c), and (d). For more modest improvements, larger sample sizes are likely, although (with the exception of the restricted procedure (d)) economy relative to the fixed sample design (a) can be anticipated whatever the magnitude of the therapeutic effect. When there is no treatment difference, designs (b) and (c) offer clear sample size reductions: see the low values of EVT in the central portions of the graphs in Figures 1(b) and (c). The triangular test (b) does not seek to distinguish between the case in which the experimental treatment and the control are equally effective and the case in which the experimental treatment is worse. Thus small samples are sufficient whenever the experimental treatment is worse. It is, however, a test against the two-sided alternative (a two-tailed test), because very early stopping on the lower boundary is unlikely if there is no treatment difference and so can lead to

**Figure 1** Some trial designs and their corresponding expected terminal amount of information (V) at termination: (**a**) fixed sample, (**b**) triangular, (**c**) double triangular, (**d**) restricted procedure.

the conclusion that the experimental treatment is significantly worse than the control. Design (b) is suitable for an experimental treatment which, due to cost or toxicity, will only be considered further if it offers a clearly demonstrated therapeutic advantage.

### The final analysis

When a sequential trial has stopped, an analysis will be conducted. A $P$-value will be calculated, and the magnitude of the treatment effect, as measured by the hazard ratio, will be estimated. A confidence interval for the hazard ratio will be found. These calculations should not use formulae developed for fixed-sample size studies. To see why not, consider a trial which has stopped because of a crossing of the upper boundary, with the conclusion that the experimental treatment is superior. Stopping *because* the experimental happens to be ahead, means that a conventional $P$-value will overstate the significance of the results, and a conventional estimate of hazard ratio will overestimate the magnitude of the treatment difference.

Underlying all conventional statistical analyses is the concept of an *ordering* of all potential datasets arising from the trial, according to the degree to which they indicate that the experimental treatment is superior to the control. We can say that a dataset which would indicate experimental superiority more strongly than another dataset according to such an ordering, is *more positive*. One particular dataset has been observed. The $P$-value $P_1$ against the one-sided alternative that the experimental treatment is superior is defined as the probability of observing a more positive dataset than the one observed by chance alone (that is when treatments are equivalent). When $P_1 < \frac{1}{2}$, it can be doubled to give the more usual $P$-value against the two-sided alternative of treatment difference. In the remainder of this section, $P$-values will concern the two-sided alternative. Estimates and confidence intervals can also be defined in terms of the ordering of potential datasets. After a fixed-sample study datasets resulting in a larger measure of advantage of experimental over control (a larger value of Z) are taken to be more positive. After a sequential trial terminating on the upper boundary, any dataset leading to earlier stopping on the upper boundary (a smaller value of V) may be regarded as more positive. The calculations used to produce the required $P$-values, estimates and confidence intervals are different from those used after a fixed-sample trial, but their meaning and interpretation are unchanged.

Figure 2 illustrates these analyses for a triangular test. The top diagram shows a particular trial outcome, with the plot of Z against V shown as a continuous path (see Section 5), ending on the upper boundary. The lower diagrams indicate the $P$- value and confidence interval for hazard ratio corresponding to this outcome. Also shown are the $P$-values and confidence intervals for all possible trial outcomes in which the upper boundary is crossed. The maximum possible value of V is denoted by $V_{max}$. Notice that if the plot of Z against V exits the triangle at the very tip (a most unlikely event) then $P$ will be equal to 0.05 and the confidence interval will have an upper limit of 1. Any earlier crossing of the upper boundary will result in $P<0.05$ and a confidence interval entirely below 1. Early crossing of the upper boundary will result in an analysis which is highly significant ($P$ very small) and in a confidence interval which although wide, is well removed from 1 (no treatment effect). Although the confidence interval is wide it does not seem ethical to allow patients to continue to be randomised to the control just to estimate with accuracy how much worse it is. Once such a large treatment difference is evident, the absolute properties of the experimental treatment remain of interest (and can be observed in an uncontrolled study), but the merits relative to control are of less interest. Later stopping results in $P$-values closer to 0.05 and to narrower confidence intervals. Exiting the triangular region at the very tip is extremely unlikely: if it were to occur then we would have $P=0.05$ and a confidence interval only slightly wider than that from an equivalent fixed sample study.

**Figure 2** Analysis of a sequential clinical trial: how the $P$-value and the 95% confidence interval for hazard ratio vary with the final amount of information.

### Implementation of sequential methods in practice

It is evident that no clinical trial will be inspected continuously, and yet the stopping rules illustrated in Figure 1 appear to presuppose this. In practice inspections of the data will occur periodically, with frequencies varying between once a week and once a year depending on the resources and need to safeguard patient safety. Figure 3 shows how a triangular test can be modified for discrete inspections. The internal boundaries, indicated by broken lines, become the operative stopping rules. Stopping is made more likely to compensate for missed opportunities for stopping between inspections. Because of their shape, the internal boundaries are called *Christmas tree boundaries*. Similar adjustments can be made to the designs shown in Figures 1(c) and (d). It is not necessary to specify in advance the timings of inspections in order to use this method, although their timings should not be influenced by the observed evidence of treatment difference inherent in the values of Z. It is not possible to draw the Christmas tree boundaries at the start of the trial as their shape emerges in response to the pattern of information available at interim analysis. In some clinical trials it is undesirable to stop very early because the large sample assumptions underlying the statistical analysis are not yet valid, and because results based on a small sample size would not be persuasive. The first inspection of the data can be delayed relative to the subsequent schedule in order to make such premature stopping impossible.

Good data flow is an essential pre-requisite for successful application of sequential methods. It is unnecessary for complete case record books to be available at each interim analysis: only details of date of entry, survival or date of death and of essential prognostic factors and treatment assignment are required. Sometimes these are completed on detachable sheets within the case record book and are transmitted and processed separately.

The methods described can be used to monitor responses other than survival times. In particular, binary (success/failure) responses, ordered (good, moderate, poor, very poor) responses, normally distributed quantitative responses and counts can all be dealt with. Randomisation to experimental and control treatments can be made in a 3:1, 2:1 or 1:2 ratio (or any other) rather than 1:1. Stratified sequential analysis, allowing for factors such as centre, stage of cancer at diagnosis or histological type of tumour, is possible. Adjustments for continuous covariates such as age or white blood count can be made.

When a clinical trial concerns a cancer which can be rapidly lethal, it is sensible to continue to recruit patients

**Figure 3** Implementation of the triangular design for discrete looks, showing the Christmas tree boundaries.

until the stopping criterion is met. Sometimes the treatment of interest will be ongoing chemotherapy. When the trial is stopped patients will no longer be required to receive study medication, and doctors are likely to alter the treatment of many of them, particularly those who were randomised to the less successful treatment. No new valid data can be accrued in such a trial. Alternatively, the treatment of interest may be applied for only a few days or weeks following randomisation. This would be the case for surgical techniques or radiotherapy. After stopping the trial, deaths would continue to be reported, from patients already recruited and still following the regimen laid down in the protocol. The trial is said to *overrun*. Trials with overrunning can be analysed within the sequential methodology. It is possible, but unlikely, that the final plotted point incorporating all of the extra data lies again within the continuation region of the study. Nevertheless the trial has stopped and it is still likely to be significant. This issue is discussed at length in Whitehead (1992*b*).

A cancer being studied might not be rapidly lethal, with median survival times being 5 years or more. In such a situation a sequential trial might be set up with a recruitment period of 3 years (say) and a follow up period of 5 years. Only in the case of a very large treatment difference apparent early during treatment would sequential monitoring lead to stopping during the recruitment period, thereby preventing some patients being exposed to a substantially inferior treatment. However, it is more likely that early stopping and publication becomes possible during the follow-up period, allowing the results to influence both clinical practice and further research years earlier than with a trial of fixed duration.

There is sometimes a reluctance to terminate a cancer clinical trial showing no sign of an advantage for the experimental treatment, or even showing a disadvantage, on the principle that 'something might turn up'. This is understandable when the biological action of the therapy is such that only long term benefit can be expected. Indeed, in the case of toxic chemotherapy, short term disadvantage can be anticipated, later to be outweighed by long term advantage. The assumption of proportional hazards mentioned at the end of the first section, would not be valid. In such a case a clear target should be set: to improve survival rates after 1 year, 2 years or maybe 5 years, (choose only one of these!). Sequential designs to detect such postponed benefit have not yet been developed although the problem is currently being explored. However, consider a situation in which no case for postponed benefit is made when the protocol is prepared. Interim results indicate no treatment difference, or worse: excess mortality on the experimental treatment. It then seems foolhardy or even callous to suggest persistence on the grounds that 'something might turn up'. It is a principle which could be maintained indefinitely, to the detriment of subjects in the study.

In some clinical trials, the research question can be stated clearly at the outset. It may be 'Does the experimental therapy reduce hazard throughout the time following treatment?', or 'Does the experimental therapy improve survival beyond 2 years after treatment?'. If the assumptions underlying the question are verified by the trial data, and the question is clearly answered, then its findings are likely to be authoritative. On the other hand, in some cases the forms of the survival patterns being compared are unclear at the outset. They will eventually be compared in many ways – overall, short-term only, long-term only, cause specific, by subgroup, and so on. If only a few of these comparisons show an advantage for the experimental treatment, then the results will be more controversial – the problems of testing multiple hypotheses suggested by the data are well-known. It may be that further trials are needed to verify hypotheses generated by the first trial.

One advantage of sequential over fixed-sample designs has already been mentioned: although validity of the power requirement in both cases depends on the proportional hazards assumption being appropriate, for sequential designs it is not necessary to know the pattern of survival on individual treatments. Furthermore, interim analyses allow the proportional hazards assumption to be checked during the trial, and perhaps to be abandoned, with a revised trial objective replacing interest in the hazard ratio. The effects of such checks on trial properties is a topic which requires methodological investigation.

Perhaps the most difficult case for a sequential design to cope with is that of a treatment with short-term survival benefit, later overturned by long-term disadvantage. If this behaviour is unforeseen, a sequential trial may stop early, recommending the new treatment on the grounds of the observed short-term benefit. However follow-up should be continued on the patients recruited, and this might motivate a further trial directed specifically at long-term effects.

A sequential design will only operate effectively if it is accepted and understood by all scientific participants in the trial. The trial statistician needs to acquaint investigators with the way in which the stopping rule is to operate, and of the consequences of the rule. Often a statistical supplement to the protocol will be prepared.

The inspections of the data could be conducted by a statistician reporting to a Data and Safety Monitoring Committee. The investigators themselves will only be told when an inspection has taken place, and whether the conclusion is to stop or continue. Emerging details of the sample path, as shown in Figure 3, will not be circulated.

It is possible that the Data and Safety Monitoring Committee may wish that the trial be terminated even before the stopping boundaries are reached, for example because of a large imbalance in the incidence of adverse events other than death. It is also possible that they see good reason for proceeding beyond the official stopping time, for example because reduced toxicity compensates for poorer survival times. Such decisions have their counterparts in the early stopping or extension of a fixed-sample clinical trial. Their ethical necessity must be allowed to override the inaccuracies they inevitably bring to the statistical analysis. The value of a sequential design, carefully discussed with the Data and Safety Monitoring Committee beforehand, is that stopping for reasons other than a boundary crossing in the sequential design is made far less likely. The sequential design is a far better model of the Committee's intentions than is a fixed-sample design.

An objection sometimes raised to the use of sequential designs is the need to specify funding requirements in advance. In fact, during the course of many fixed-sample trials, low accrual rates, high drop-out rates and unexpected delays and complications can add to both duration and cost. Perhaps the real difficulty is in facing up to these uncertainties from the outset.

Implementation of sequential methods in clinical trials is greatly eased by use of a computer program such as PEST (Planning and Evaluation of Sequential Trials: Whitehead and Brunier, 1989). The necessary calculations for design, monitoring and analysis, for survival studies and for other response types are all included.

### Alternative approaches to sequential investigation

Extensive reviews of sequential methodology suitable for clinical research are given in Chapter 6 of Whitehead (1992*a*) and by Jennison and Turnbull (1990). The earliest methods to be applied extensively in clinical trials were the 'group sequential' methods of Pocock (1977) and of O'Brien and Fleming (1979). Pocock (1982) discusses improvements to his 1977 procedure, but these seem to have been overlooked by practitioners. These methods require that inspections of the data are conducted according to a regular pattern. In a survival study this means that the number of new deaths observed at each inspection is to be a constant. More flexible extensions of the 'group sequential' approach are provided by the 'α-spending function' method of Lan and DeMets (1983).

A completely different view of the philosophy of inferential statistics as a whole is provided by the Bayesian school. Bayesian analyses do not quote *P*-values or confidence limits, but instead make probability statements concerning unknown parameters such as the hazard ratio. Such probability statements combine subjective opinions held prior to the collection of clinical data, with the results of the trial. One consequence of the Bayesian approach is that their analyses are not affected by the use of stopping rules. Bayesian sequential designs are described by Berry (1989) and Freedman and Spiegelhalter (1989) and are discussed by Whitehead (1993).

### Conclusion

In this paper the principal objectives and methods of sequential clinical trials have been described. The work presented has been motivated by the natural attractions of stopping trials as soon as the data are sufficient, and in response to valid criticisms of the oversimplified nature of early attempts to construct sequential designs. Problems still remain with many types of clinical trial, including those with more than two treatments and those in which the *monitoring* of more than one endpoint is essential. (The *analysis* of more than one endpoint in a trial stopped due to the monitoring of just one of them is a different problem for which some limited results are already available).

Despite the limitations mentioned above, sequential methods do provide potential designs for a wide class of clinical trials in cancer. Retrospective consideration of how the methods would have affected completed trials has already been made by Rosner and Tsiatis (1989) (see also Facey & Whitehead, 1990), and by Donaldson *et al.* (1993). In both papers a worthwhile reduction in sample size without a change in trial conclusions was claimed. The development of sequential methods has now reached the stage at which extensive exploration of their use in cancer trials is appropriate.

In this paper attention has been restricted to individual clinical trials. Often more than one clinical trial of the same novel treatment is considered necessary either to obtain registration of a drug or to alter clinical opinion and practice. Here, conventional power requirements have been used to set a flexible stopping rule rather than a rigid sample size. Issues of what level of significance to specify and how to plan and combine multiple clinical trials affect sequential and non-sequential designs alike. Exciting new methodologies which combine sequential concepts with those of meta-analysis will be needed to decide when to stop performing new studies of the same medical treatment, and how to monitor more than one ongoing trial addressing the same therapeutic question.

This work is supported by MRC Project Grant G900 8019. I am grateful to Dr A.N. Donaldson, Mr R.R. Hall, Dr D. Machin, Mr A. Ritchie, Mr P.H. Smith, Professor R.L. Souhami and an anonymous referee for helpful and constructive comments made on an earlier draft of this paper.

### References

ARMITAGE, P. (1975). *Sequential Medical Trials*, (2nd Edition). Oxford: Blackwell.

BERRY, D.A. (1989). Monitoring accumulating data in a clinical trial. *Biometrics*, **45**, 1197–1211.

DONALDSON, A.N., WHITEHEAD, J., STEPHENS, R. & MACHIN, D. (1993). A simulated sequential analysis based on data from two MRC trials. (Submitted to the *Br. J. Cancer*).

FACEY, K.M. & WHITEHEAD, J. (1990). Letter to the Editor. *Statist. Med.*, **9**, 853–854.

FREEDMAN, L.S. & SPIEGELHALTER, D.J. (1989). Comparison of Bayesian with group sequential methods for monitoring clinical trials. *Controlled Clin. Trials*, **10**, 357–367.

GELLER, N.L. & POCOCK, S.J. (1987). Interim analysis and randomised clinical trials: ramifications and guidelines for practitioners. *Biometrics*, **43**, 213–223.

JENNISON, C. & TURNBULL, B.W. (1990). Statistical approaches to interim monitoring of medical trials: A review and commentary. *Statistical Sci.*, **5**, 299–317.

JONES, D.R., NEWMAN, C.E. & WHITEHEAD, J. (1982). The design of a sequential clinical trial for the comparison of two lung cancer treatments. *Statist. Med.*, **1**, 73–82.

KILPATRICK, G.S. & OLDHAM, P.D. (1954). Calcium chloride and adrenaline as bronchial dilators compared by sequential analysis. *Br. Med. J.*, **ii**, 1388–1391.

LAN, K.K.G. & DEMETS, D.L. (1983). Discrete sequential boundaries for clinical trials. *Biometrika*, **70**, 659–663.

MACHIN, D. (1992). Interim analysis and ethical issues in the conduct of trials. In: Williams, C.J. (ed.) *Introducing New Treatments for Cancer: Practical, Ethical and Legal Problems*. Wiley. 203–215.

MEDICAL RESEARCH COUNCIL UROLOGICAL WORKING PARTY. (1991). Metastatic renal carcinoma. A randomised trial of α-Interferon *vs* Medroxyprogesterone Acetate. Study Protocol RE01, MRC Cancer Trials Office, Cambridge.

MONTANER, J.S.G., LAWSON, L.M., LEVITT, N., BELZBERG, A., SCHECHTER, M.T. & RUEDY, J. (1990). Corticosteroids prevent early deterioration in patients with moderately severe *pneumocystis carinii* pneumonia and the acquired immunodeficiency syndrome (AIDS). *Ann. Intern. Med.*, **113**, 14–20.

NEWMAN, C.E., COX, R., FORD, C.H.J., JOHNSON, J.R., JONES, D.R., WHEATON, M. & WHITEHEAD, J. (1985). Reduced survival with radiotherapy and razoxane compared with radiotherapy alone for inoperable lung cancer in a randomised double-blind trial. *Br. J. Cancer*, **51**, 731–732.

O'BRIEN, P.C. & FLEMING, T.R. (1979). A multiple testing procedure for clinical trials. *Biometrics*, **35**, 549–556.

PEACE, K.E. (1992). (ed.). *Biopharmaceutical Sequential Statistical Applications*. New York: Marcel Dekker, Inc.

POCOCK, S.J. (1977). Group sequential methods in the design and analysis of clinical trials. *Biometrika*, **64**, 191–199.

POCOCK, S.J. (1982). Interim analyses for randomised clinical trials: the group sequential approach. *Biometrics*, **38**, 153–162.

POCOCK, S.J. (1992). When to stop a clinical trial. *Br. Med. J.*, **305**, 235–240.

ROSNER, G.L. & TSIATIS, A.A. (1989). The impact that group sequential tests would have made on ECOG clinical trials. *Statist. Med.*, **8**, 505–516.

STORB, R., DEEG, J., WHITEHEAD, J., APPELBAUM, F., BEATTY, P., BENSINAER, W., BUCKNER, C.D., CLIFT, R., DONEY, K., FAREWELL, V., HANSEN, J., HILL, R., LUM, L., MARTIN, P., MCGUFFIN, R., SANDERS, J., STEWART, P., SULLIVAN, K., WITHERSPOON, R., YEE, G. & THOMAS, E.D. (1986). Methotrexate and cyclosporine compared with cyclosporine alone for prophylaxis of acute graft versus host disease after marrow transplantation for leukemia. *N. Engl. J. Med.*, 314, 729–735.

WHITEHEAD, J. (1992*a*). *The Design and Analysis of Sequential Clinical Trials (2nd Edition)*. Ellis Horwood: Chichester.

WHITEHEAD, J. (1992*b*). Overrunning and underrunning in sequential clinical trials. *Controlled Clinical Trials*, **13**, 106–121.

WHITEHEAD, J. (1993). The case for frequentism in clinical trials. *Statist. Med.* (in press).

WHITEHEAD J. & BRUNNER, H. (1989). *PEST 2.0 Operating Manual.* Reading University.

WHITEHEAD, J., JONES, D.R. & ELLIS, S.H. (1983). The analysis of a sequential clinical trial for the comparison of two lung cancer treatments. *Statist. Med.*, **2**, 183–190.

**Appendix:** Survival probabilities and hazard ratios.

Denote by $P_C$ and $P_E$ respectively, the percentages of patients surviving for longer than six months on the control and experimental treatments respectively. Denote the hazard ratio by HR. Let the Greek letter θ denote minus the (natural) logarithm of HR. That is

$$\theta = -\log \mathrm{HR}. \quad (1)$$

When hazards are proportional, θ is also given by

$$\theta = -\log\{-\log(P_E/100)\} + \log\{-\log(P_C/100)\}, \quad (2)$$

where all logs are natural. In fact, θ is given by equation (2) whatever time period is specified, and not just for the case of '6 months' used above. This lack of dependence on the time period is a consequence of the proportional hazards assumption.

For $P_C = 50$ and $P_E = 65$, equation (2) gives $\theta = 0.48$, and for $\mathrm{HR} = 0.62$, equation (1) also gives $\theta = 0.48$ confirming the equivalence of the specifications given in Section 2.